# The Swedish Exercise Self-Efficacy Scale (ESES-S): reliability and validity in a rheumatoid arthritis population

**DOI:** 10.3109/09638288.2014.998780

**Published:** 2015-09-04

**Authors:** Thomas Nessen, Ingrid Demmelmaier, Birgitta Nordgren, Christina H. Opava

**Affiliations:** ^a^ Division of Physiotherapy, Department of Neurobiology, Care Sciences and Society, Karolinska Institutet, Stockholm, Sweden; ^b^ Department of Rheumatology, Karolinska University Hospital, Stockholm, Sweden

**Keywords:** Internal consistency, measurement properties, physical activity, questionnaire, rehabilitation, rheumatology, test–retest

## Abstract

*Purpose*: The aim of the present study was to investigate aspects of reliability and validity of the Exercise Self-Efficacy Scale (ESES-S) in a rheumatoid arthritis (RA) population. *Methods*: A total of 244 people with RA participating in a physical activity stkudy were included. The six-item ESES-S, exploring confidence in performing exercise, was assessed for test–retest reliability over 4–6 months, and for internal consistency. Construct validity investigated correlation with similar and other constructs. *Results*: An intraclass correlation coefficient (ICC) of 0.59 (95% CI 0.37–0.73) was found for 84 participants with stable health perceptions between measurement occasions. Cronbach’s alpha coefficients of 0.87 and 0.89 were found at the first and second measurements. Corrected item-total correlation single ESES-S items ranged between 0.53 and 0.73. Construct convergent validity for the ESES-S was partly confirmed by correlations with health-enhancing physical activity and outcome expectations respectively (Pearson’s *r* = 0.18, *p* < 0.01). Construct divergent validity was confirmed by the absence of correlations with age or gender. No floor or ceiling effects were found for ESES-S. *Conclusions*: The results indicate that the ESES-S has moderate test–retest reliability and respectable internal consistency in people with RA. Construct validity was partially supported in the present sample. Further research on construct validity of the ESES-S is recommended.

**Implications for Rehabilitation**
- Physical exercise is crucial for management of symptoms and co-morbidity in rheumatoid arthritis.
- Self-efficacy for exercise is important to address in rehabilitation as it regulates exercise motivation and behavior.
- Measurement properties of self-efficacy scales need to be assessed in specific populations and different languages.

## Introduction

The positive effects of physical activity for individual and population health are well documented [1]. Rheumatoid arthritis (RA) is an inflammatory auto-immune disease, and physical activity forms a crucial part of its management [2–4]. However, the RA population performs less physical activity than recommended for a healthy lifestyle, and has difficulties in maintaining health-enhancing physical activity (HEPA) levels over time [5–7]. People with RA experience higher levels of activity limitation, pain and fatigue, and lower levels of perceived health than the general population [8–10], and these factors are associated with physical inactivity in the RA population [5,11].

To be able to understand why some people regularly participate in activities adequate to improve physical fitness and health, and some do not, it is crucial to identify determinants relevant for an individual’s decision to initiate, adopt and maintain physical activity [12,13]. One psychological mediator of physical activity is self-efficacy [14,15], defined as peoples’ judgments of their capabilities to organize and apply courses of action that are required to produce given attainments [16]. It operates to regulate human motivation, behavior and well-being. Furthermore, self-efficacy influences other determinants such as goals and aspirations [17], and is associated with psychological determinants of physical activity such as fear-avoidance [18], outcome expectations [17,19,20] and anxiety/depressive states [21]. Another important aspect of self-efficacy is that it is not general in nature but related to specific situations, and also temporary, task-related, and relatively easy to influence [16]. One of several ways to measure self-efficacy is the Exercise Self-Efficacy Scale (ESES), a self-administered questionnaire developed in English by Dzewaltowski [12], and adapted to Swedish (ESES-S) by Denison et al. [22,23]. The ESES evaluates self-efficacy to perform exercise despite a number of common barriers. As the ESES was originally developed to assess self-efficacy in a general population [12], one crucial issue is whether this scale suits the characteristics of different subpopulations [24], such as people with RA. In addition, versions of the scale in different languages have to be assessed for measurement properties. Internal consistency and test–retest reliability have previously been reported with satisfactory results in other subpopulations for the ESES, in both the English [12,25] and Swedish versions [23]. However, to our knowledge the ESES-S has not previously been tested for reliability and validity in people with RA.

### Aim

The aim of the present study was to investigate aspects of reliability and validity of the ESES-S in an RA population.

## Materials and methods

### Participants

A convenience sample of 244 people with RA participating in the Physical Activity in RA (PARA) 2010 study was included [26]. The inclusion criteria were: RA according to the American College of Rheumatology (ACR) criteria [27], 18–75 years and independent in daily activities measured by Stanford Health Assessment Questionnaire Disability Index (HAQ) scores ≤2 [26]. The participants were identified from six rheumatology clinics in Sweden. They did not reach the recommendations on physical activity for a healthy lifestyle, and did not have any other health condition that prevented HEPA. They were also speaking and understanding the Swedish language.

### Data collection

All data were collected by questionnaires at two measurement occasions before a planned physical activity intervention. More information on the PARA 2010 study is described elsewhere [26]. The first measurement was performed in the recruitment phase of the PARA 2010 study, and the second measurement was performed at baseline immediately before the start of the intervention. The time interval between the first and second measurements varied between four and six months, as the intervention started at different times at the participating study sites.

### Assessment methods

Exercise self-efficacy was measured with the self-administered ESES-S. The main question “How confident are you to exercise …” is followed by six items describing common barriers for exercise: “in spite of your work schedule”, “when physically fatigued”, “when exercise is boring”, “with minor injuries”, “in spite of other time demands”, “in spite of family responsibilities” [12]. The original version of the ESES employs a 0–100 scale, but the Swedish version ranges from “not certain” (=1) to “very certain” (=10), thus retaining a similar scale structure as the original. A total score (6–60) for the six items is calculated.

The modified Fear Avoidance Belief Questionnaire (mFABQ) focuses on peoples’ beliefs about how physical activity affects their current pain, and is mainly based on fear theory and fear-avoidance cognitions [28]. The mFABQ comprises four items scored on 0–6 scales, where 0 signifies absence of beliefs in relation between pain and physical activity, and 6 signifies a strong belief. A total score (0–24) is calculated.

Both HEPA and exercise were measured using the International Physical Activity Questionnaire (IPAQ) short version [29,30]. HEPA was assessed with an aggregated dichotomized score indicating whether HEPA levels were reached or not, and exercise measured with the total minutes per day of estimated vigorous physical activity during the previous 7 days.

Outcome expectations for physical activity were measured with two questions: “How certain are you that HEPA is beneficial for your health in the long run?” and “How certain are you that HEPA has a positive impact on your RA-related difficulties?”. The questions are measured on scales 1–10 where 1 signifies “not at all sure” and 10 “totally sure” [26].

General health perception, pain and fatigue were rated on 0–100 mm visual analogue scale (VAS) where 0 signifies the best condition and 100 the worst.

Activity limitation was assessed with the HAQ [31] employing a 0–3 scale, where 0 signifies performing tasks without any problems and 3 that it is impossible to perform tasks. The HAQ has eight items, and an average (0–3) for all items is calculated.

Perception of a depressive state was measured by one item in the EQ-5D questionnaire [32,33] using an 1–3 scale with 1 being indicative of not worried or depressed, and 3 of worried or depressed to a high degree.

### Data management and statistical procedure

Due to the interval between the two measurement occasions in the test–retest investigation, participants were excluded if perceived general health, pain or fatigue differed more than 20 mm on a 100 mm VAS, or if EQ-5D differed 1 step or more on a 1–3 scale. The exclusions were made to protect from bias in terms of health changes between measurement occasions that were likely to affect the ESES scores.

The intraclass correlation coefficient (ICC) was used to assess test–retest reliability, comparing the results of the first and second measurement occasion. A two-way mixed calculation with absolute agreement, and variability presented as 95% confidence interval, was used. Agreement was classified as follows: ICC ≤ 0.4 = poor; 0.4 to ≤0.8 = moderate; and ≥0.8 = good [34].

Internal consistency was calculated with Cronbach’s alpha to measure the degree to which single ESES-S items measured a common construct, and to assess the correlation between each item and the sum of the other items (corrected item-total correlation). For measuring common construct, alpha coefficients between 0.7 and 0.8 would be considered as minimally acceptable, 0.8–0.9 as respectable and over 0.9 as very good [35–37]. For the corrected item-total correlation alpha coefficients over 0.4 are considered good [38].

In order to assess validity of the ESES-S in an RA population, we formulated a number of hypotheses based on previous research on self-efficacy. For construct convergent validity, it was thus hypothesized that the ESES-S should correlate positively with HEPA, exercise [39,40] and outcome expectations on physical activity [40]. ESES-S should also correlate negatively with pain, activity limitation [41,42] and fear avoidance beliefs [43]. For construct divergent validity, the ESES-S should have no correlation with age or gender. To calculate validity, the Pearson correlation coefficient (*r*) was used and categorized as follows: 0.0–0.2 is a very weak relationship, 0.2–0.4 is weak, 0.4–0.6 is moderate, 0.6–0.8 is strong and 0.8–1.0 is very strong [44]. Point-biserial correlation is a special case of the Pearson correlation coefficient, and was used to calculate the relation between dichotomized and interval variables [45].

Ceiling and floor effects were examined for the ESES-S single items, as the percentage of individuals selecting the highest and lowest scores respectively. For the ESES-S total score, the percentage of individuals assigned to any of the 10 highest and 10 lowest scores respectively were used. Over 15% responses on the highest or lowest scores were considered to represent ceiling or floor effects [24].

All calculations, except that of test–retest reliability, were based on the data from the first measurement occasion. The Statistical Package for the Social Sciences (SPSS) version 22 (Armonk, NY) was used for the statistical analysis.

### Ethics

Ethical approvals were obtained from the Stockholm regional ethical review board (2010/1232-31/1, 2011/1241-32). The participants gave their informed written consent by filling out and returning their postal questionnaires.

## Results

Ninety-two participants had stable health conditions between the two measurement occasions, and were thus included in the test–retest sample. They were overall exhibiting similar characteristics to the total study sample, with some differences on perceived health, pain and fatigue (Table 1).

**Table 1.** Demographic data for the total study sample (*n* = 244) and the test–retest subsample (*n* = 92).

	Total study sample	Missing, *n*	Test–retest subsample	Missing, *n*
Age, years, mean (SD)	59 (9.0)		59 (8.7)	
Females, *n* (%)	198 (81.1)		74 (80.4)	
Income below average, *n* (%)	75 (30.9)	2	23 (25.0)	
Other diagnoses, *n* (%)	141 (57.8)	2	49 (53.3)	
Perceived health, 1–100, mean (SD)	31 (33.8)	1	22 (22.8)	
Pain, 1–100, mean (SD)	29 (25.0)		19 (20.9)	
Fatigue, 1–100, mean (SD)	36 (39.9)		26 (24.1)	
ESES total, 6–60, mean (SD)	32 (12.3)	12	32 (12.3)	2
mFABQ total, 0–24, mean (SD)	6.3 (4.9)	1	5.3 (4.5)	1
HEPA obtained, *n* (%)	188 (77.0)		74 (80.4)	
Outcome expectations on health, 1–10, mean (SD)	9.4 (1.3)		9.6 (1.3)	
Outcome expectations on RA-related difficulties, 1–10, mean (SD)	7.9 (2.4)		7.8 (2.5)	
HAQ total score, 0–3, mean (SD)	0.54 (0.55)	2	0.44 (0.52)	1
EQ-5D, 1–3, mean (SD)	1.4 (0.5)		1.2 (0.4)	

In the test–retest investigation, eight of the 92 individuals had missing data in the first or second measurement occasion, and thus calculations of test–retest reliability for the ESES-S total score was performed for the remaining 84 participants. The ICC was 0.59 (95% CI, 0.37–0.73) for the ESES total score, and 0.45 (CI 0.17–0.64, *n* = 86), 0.42 (CI 0.13–0.62, *n* = 89), 0.43 (CI 0.13–0.62, *n* = 88), 0.67 (CI 0.50–0.79, *n* = 89), 0.61 (CI 0.40–0.74, *n* = 87), 0.56 (CI 0.33–0.71, *n* = 87) for items 1–6 respectively. The results thus indicated “moderate” agreement for ESES-S between measurement occasions 1 and 2. Cronbach’s alpha was 0.87 at the first measurement occasion and 0.89 at the second, indicating that the ESES-S had “respectable” inter-relatedness of items. Item-total correlations for the ESES-S single items are presented in Table 2, indicating a “good” corrected item-total correlation. The ESES-S total score for the 84 participants had a mean of 32 (CI, 29–34) at the first measurement occasion, and 35 (CI, 32–37) at the second.

**Table 2.** Corrected item-total correlations for the six items in ESES-S.

How confident are you to exercise	Cronbach’s *α*
1. In spite of your work schedule	0.69
2. When physically fatigued	0.73
3. When exercise is boring	0.53
4. With minor injuries	0.64
5. In spite of other time demands	0.71
6. In spite of family responsibilities	0.68

In preparation for the calculation of construct validity, an initial screening revealed skewed distributions of the two questions on outcome expectation (Table 1). Subsequently they were dichotomized into “not certain” (ratings = 1–9), and “certain” (rating = 10). The results of the construct validity investigation are shown in Table 3. Construct convergent validity was partly supported through significant, although weak, correlation between the ESES-S and HEPA, as well as outcome expectations regarding positive impact on RA-related difficulties. Divergent construct validity was supported by the absence of correlations with age and gender.

**Table 3.** Correlations between the ESES-S total score and other study variables.

	Age^a^	Gender^b^	General pain (VAS)	HAQ total score	HEPA^a,b^	Exercise	Outcome expectations on health^b^	Outcome expectations on RA-related difficulties^a,b^	mFABQ total score
ESES-S									
Pearson correlation	0.05	−0.06	−0.07	−0.08	0.18*	0.03	0.12	0.18*	−0.07
Sig. (2-tailed)	0.46	0.36	0.31	0.25	0.01	0.62	0.08	0.01	0.29
*N*	232	232	232	230	232	232	232	232	231

^a^Results in line with pre-set hypotheses. ^b^Point-biserial calculations were applied. *Correlation is significant at the 0.01 level (2-tailed).

No floor or ceiling effects for the ESES-S were found.

## Discussion

This study provides new knowledge on the measurement properties of the ESES-S in people with RA. The ESES-S demonstrated moderate test–retest reliability. Internal consistency was respectable, as was item-total correlation. Construct convergent and divergent validity were only partially supported by correlations in line with our pre-set hypotheses. Furthermore, the ESES-S showed no ceiling or floor effects.

Test–retest reliability investigation provided an ICC of *r* = 0.59, which was lower than those previously reported by Yordy (*r* = 0.76) and Johansson (*r* = 0.64) for the original English and Swedish ESES respectively [23,25]. Nevertheless, these results were all moderate according to the adapted ICC classification of agreement [34]. Despite excluding people with major changes in health status from the test–retest analysis, the present results may have been influenced by the long time interval of four to six months between the two measurement occasions. A time interval of 2–14 days is a common recommendation [46], even though there are no absolute limits for time intervals between measurement occasions [47]. Hence, it should be prudent to expect changes in the individual and the environment over time [46]. On the other hand, due to the dynamic nature of self-efficacy, changes may also occur over hours, days or weeks [48]. One possible advantage of a long test–retest interval is that sensitization to the questions may be reduced [46]. This may lead to less risk of remembering the questions and responses from the previous measurement occasion. Another possible bias is that, at the second measurement occasion, the participants had committed to participate in a physical activity intervention. Even though the results did not yield any significant differences in ESES-S total score means between the measurement occasions, such commitment may lead to changes in self-efficacy. These changes can occur through participants inquiring about more information on exercise, observing others exercising or performing exercise to a higher extent than if not committed to an intervention. Internal consistency was respectable with alpha coefficients of 0.87 and 0.89, indicating acceptable interrelatedness between the ESES-S items. This is in line with previous results for the ESES in other populations. Yordy tested the internal consistency of the ESES in a sample of college students, which yielded an alpha coefficient of 0.83 [25] and Johansson found an alpha coefficient for the ESES-S of 0.85 in patients with low back pain [23].

Construct convergent and divergent validity were only partially supported, which may question the validity of the ESES-S in the present sample. However, it may also indicate that our hypotheses, although founded in theory and previous research, could be incorrect or incomplete. For example, other variables, such as fatigue, are also known to correlate with self-efficacy [49] and could have been used to test the ESES-S validity in the present study. Furthermore, previous research is inconclusive as to correlations between ESES and other variables. For instance, even though there is a substantial body of research indicating a correlation between self-efficacy and physical activity [39], some studies report absence of such correlation [50]. Interestingly, HEPA was found in the present study to have a weak, but statistically significant, positive correlation with the ESES-S, while planned and structured exercise was not. This could indicate that people with RA perceive the concept of exercise more in line with the broader concept of physical activity. It should, however, also be noted that the HEPA variable was dichotomized, which may result in loss of strength in the correlation [51].

One reason for the hypothesized correlations of the present study not being supported, could be that self-reports may contain systematic bias. If all variables share a common data collection method, in this case self-reports, a potential contamination of the correlations between the variables may occur [52]. Another possible bias is response distortion, such as response styles biased in some direction, for example overly positive responses. A third bias could be distorted response sets, where the respondent tries to make an impression, for example to respond in a socially desirable way [46,53]. In spite of the limitations of self-reports, they are relevant to measure many constructs, and self-report instruments are common in social and behavioral sciences [54]. However, since all variables in the present study were self-reports, it could be useful to complement them with other methods such as objective measures, for example physical activity monitoring with accelerometers, to reduce potential bias [52].

The external validity extends to people with RA fulfilling the PARA 2010 criteria [26], rather than to the RA population as a whole. Hence, the participants were not reaching HEPA at the inclusion of the study, and they had no diseases preventing them from reaching HEPA. Moreover, they had expressed interest in attending an intervention with organized exercise, which is likely to indicate a more positive attitude toward HEPA compared to the RA population as a whole.

In conclusion, our results suggest that the ESES-S has moderate test–retest reliability and respectable internal consistency. Construct convergent and divergent validity were only partially supported. Further research on the concurrent construct validity, as well as on the predictive validity, of the ESES-S is recommended. Moreover, using complementing methods such as objective measures and adjusting the hypotheses for self-efficacy correlations by including other variables associated with self-efficacy, may be appropriate before using it in research and clinical environments.
